# A Compound of Obstetrics

**Published:** 1887-05

**Authors:** 


**A Compend of Obstetrics.** Especially adapted to the use of medical students and physicians. By Henry G. Landis, A. M., M. D. Third edition, thoroughly revised, with new illustrations. Philadelphia: P. Blakiston, Son & Co., 1012 Walnut street. 1887.

This compend is one of a series of compends designed to assist students in preparing for examinations, bringing out, in a short compass, the more important points or facts in the special subject of which it treats. We think such works can be of essential service, and the author has judiciously fulfilled the object for which the work was undertaken.

